# Inhibition of Tyrosine-Phosphorylated STAT3 in Human Breast and Lung Cancer Cells by Manuka Honey is Mediated by Selective Antagonism of the IL-6 Receptor

**DOI:** 10.3390/ijms20184340

**Published:** 2019-09-05

**Authors:** Priyanka Aryappalli, Khadija Shabbiri, Razan J. Masad, Roadha H. Al-Marri, Shoja M. Haneefa, Yassir A. Mohamed, Kholoud Arafat, Samir Attoub, Otavio Cabral-Marques, Khalil B. Ramadi, Maria J. Fernandez-Cabezudo, Basel K. al-Ramadi

**Affiliations:** 1 Department of Medical Microbiology & Immunology, College of Medicine and Health Sciences, United Arab Emirates University, Al Ain, United Arab Emirates; 2 Department of Biochemistry, College of Medicine and Health Sciences, United Arab Emirates University, Al Ain, United Arab Emirates; 3 Department of Pharmacology & Therapeutics, College of Medicine and Health Sciences, United Arab Emirates University, Al Ain, United Arab Emirates; 4 Department of Immunology, Institute of Biomedical Sciences, University of São Paulo, São Paulo, SP 05508-000, Brazil; 5 Harvard-MIT Health Sciences and Technology Division, Massachusetts Institute of Technology, Cambridge, MA 02139, USA

**Keywords:** Manuka honey, Flavonoids, IL-6 receptor, STAT3, IL-6, breast and lung cancers

## Abstract

Aberrantly high levels of tyrosine-phosphorylated signal transducer and activator of transcription 3 (p-STAT3) are found constitutively in ~50% of human lung and breast cancers, acting as an oncogenic transcription factor. We previously demonstrated that Manuka honey (MH) inhibits p-STAT3 in breast cancer cells, but the exact mechanism remained unknown. Herein, we show that MH-mediated inhibition of p-STAT3 in breast (MDA-MB-231) and lung (A549) cancer cell lines is accompanied by decreased levels of gp130 and p-JAK2, two upstream components of the IL-6 receptor (IL-6R) signaling pathway. Using an ELISA-based assay, we demonstrate that MH binds directly to IL-6Rα, significantly inhibiting (~60%) its binding to the IL-6 ligand. Importantly, no evidence of MH binding to two other cytokine receptors, IL-11Rα and IL-8R, was found. Moreover, MH did not alter the levels of tyrosine-phosphorylated or total Src family kinases, which are also constitutively activated in cancer cells, suggesting that signaling via other growth factor receptors is unaffected by MH. Binding of five major MH flavonoids (luteolin, quercetin, galangin, pinocembrin, and chrysin) was also tested, and all but pinocembrin could demonstrably bind IL-6Rα, partially (30–35%) blocking IL-6 binding at the highest concentration (50 μM) used. In agreement, each flavonoid inhibited p-STAT3 in a dose-dependent manner, with estimated IC_50_ values in the 3.5–70 μM range. Finally, docking analysis confirmed the capacity of each flavonoid to bind in an energetically favorable configuration to IL-6Rα at a site predicted to interfere with ligand binding. Taken together, our findings identify IL-6Rα as a direct target of MH and its flavonoids, highlighting IL-6R blockade as a mechanism for the anti-tumor activity of MH, as well as a viable therapeutic target in IL-6-dependent cancers.

## 1. Introduction

The characteristics that define successful cancers include not only the capacity for sustained, self-sufficient proliferation, but also increased resistance to apoptosis, metabolic reprogramming, acquisition of migration and invasion capabilities, and pro-angiogenic potential [1]. In this context, human triple-negative breast cancers (TNBCs) represent a major challenge in treatment owing to their inherent resistance to chemotherapy and high capacity for metastatic spread [2,3]. Similarly, non-small cell lung cancer (NSCLC), which accounts for the great majority (~85%) of all lung cancers, is relatively resistant to chemotherapy and is associated with poor prognosis, with an expected survival of fewer than two years in patients with advanced disease [4].

Interleukin-6 (IL-6) is a proinflammatory cytokine with pleiotropic functions in regulating the growth and differentiation of different types of cancer cells, including breast, colon, and lung cancers [5,6,7]. The binding of IL-6 to its receptor, IL-6Rα, triggers a heterodimeric association with the signal-transducing receptor gp130 to form a signaling complex that initiates the phosphorylation and activation of Janus kinases JAK1 and JAK2 [8]. This catalyzes the tyrosine phosphorylation of the transcription factor STAT3 (signal transducer and activator of transcription 3), which dimerizes and translocates to the nucleus, thereby initiating a complex transcriptional set that promotes cell growth and inhibits apoptosis [9]. 

High levels of IL-6 are expressed in malignant breast cancers where, together with breast stromal fibroblasts, drive both autocrine and paracrine growth through the IL-6/IL-6R/STAT3 positive feedback loop [10,11,12]. IL-6 has also been implicated in the malignant transformation of breast cancer stem cells and in the enhancement of cancer cell metastatic potential and epithelial to mesenchymal transition [13,14,15]. Similarly, patients with lung adenocarcinoma usually have elevated levels of IL-6, which are associated with poor prognosis [16,17,18]. Constitutively, activated tyrosine (Y705)-phosphorylated STAT3 (p-STAT3) has been demonstrated in many human breast [12] and lung [7] cancer cell lines, in 40–50% of primary human breast cancers [12,19], and 22–65% of non-small cell lung cancers [20], making it an attractive target for the development of anti-cancer therapies. The induction of STAT3 transcriptional activity increases the expression of many genes involved in cancer cell proliferation, survival, migration, invasion, angiogenesis, and metastasis [21]. Given the central role of the IL-6-STAT3 pathway in the regulation of breast and lung cancer progression and metastasis, blockade of its various components may potentially lead to new therapeutic modalities. 

Previous work from our group demonstrated the potential use of manuka honey (MH) as a modulatory anti-cancer agent [22,23]. The treatment of human breast cancer cells with MH led to a dose and time-dependent inhibition of the transcriptional activity of STAT3 [23]. The potency of MH in inhibiting this critical signaling pathway in cancer cells was demonstrated by the fact that as low as 0.03% solution (w/v) of MH (equivalent to a concentration of 0.3mg/mL) was sufficient to cause a significant reduction in p-STAT3 levels [23]. Importantly, inhibition of p-STAT3 was accompanied by a reduction in IL-6 secretion, hence depriving breast cancer cells of this critical growth-promoting factor. In this context, MH was able to inhibit the migration, invasion and angiogenic potential of breast cancer cells. These findings identify multiple functional pathways affected by MH in human breast cancer and highlight the IL-6/STAT3 signaling pathway as a potentially critical target in this process [23]. However, the precise mechanism by which MH inhibits p-STAT3 activation remains to be elucidated. 

In addition to the IL-6/STAT3 pathway, the diverse properties of cancer cells are regulated by signaling through multiple receptors, including platelet-derived growth factor receptor (PDGF-R), epidermal growth factor receptor (EGF-R), fibroblast growth factor receptor (FGF-R), vascular endothelial growth factor receptor (VEGF-R) and insulin-like growth factor-1 receptor (IGF-1R). Src family kinases are critical mediators in all of these receptor-signaling pathways and play an important role in tumor resistance [24,25]. Moreover, Src kinases are intricately involved in integrin signaling, thereby regulating tumor metastasis [26,27]. STAT activation is also known to be induced by growth factor receptors, such as EGF-R, and the Src family of kinases, particularly c-Src [28]. Given the central role played by c-Src in mediating signaling from a multitude of other receptors on cancer cells, the potential effect of MH on Src activity remains unknown.

In the present study, we demonstrate that MH-induced inhibition of oncogenic p-STAT3 in human TNBC cell line MDA-MB-231 and NSCLC cell line A549 is associated with decreased levels of gp130 and p-JAK2 proteins, two critical upstream components of the IL-6R signaling pathway. Importantly, MH had no effect on p-Src levels in both cancer cell lines. Furthermore, using recombinant proteins, we demonstrate that MH binds directly and specifically to IL-6Rα, interfering with the binding of IL-6 ligand. Thus, we identify the IL-6Rα chain as a direct target of MH. Finally, molecular docking studies identified potential binding sites of MH flavonoids on IL-6Rα. Our findings represent the first demonstration of the ability of MH to act as an antagonist of a key pro-oncogenic pathway through binding to the IL-6Rα protein expressed by human breast and lung cancer cells.

## 2. Results

### 2.1. MH-Induced Loss of p-STAT3 is Associated with Decreased gp130 and p-JAK2

**Figure 1:**
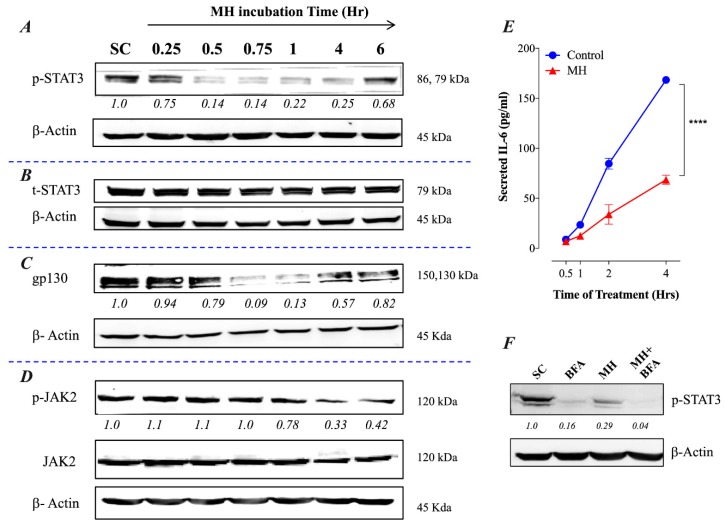
MH inhibits the IL-6 receptor signaling pathway in breast cancer cells. MDA-MB-231 cells were exposed to 1% MH, or an equivalent sugar control (SC) solution for the indicated times and analyzed by Western blotting for the relative protein expression of tyrosine-phosphorylated p-STAT3 (**A**), total STAT3 (**B**), gp130 (**C**), and JAK2 / p-JAK2 (**D**). β-actin was used as a loading control. (**E**) Analysis of IL-6 secretion in MDA-MB-231 cells by ELISA. MDA-MB-231 cells were exposed to 1% MH for 0.5, 1, 2, and 4 h, following which cell-free supernatants were collected and analyzed for IL-6 content by ELISA. Asterisks denote statistically significant differences in IL-6 levels of the experimental group compared to control (**** *p* < 0.0001), as determined by two-way ANOVA. (**F**) Effect of Brefeldin A (1 mg/mL) on the expression of p-STAT3 in MDA-MB-231 cells. The cells were exposed to Brefeldin A alone or with 1% MH for 4 h and analyzed for the expression of p-STAT3. As a control, cells were exposed to 1% SC solution and analyzed. The numbers below each blot indicate changes in band intensity compared to controls, as determined by densitometric analysis. The data are representative of 2–3 independent experiments.

We have recently demonstrated that MH affects multiple functions of breast cancer cells through the inhibition of p-STAT3 functional activity and IL-6 secretion [23]. As we have previously shown, inhibition of p-STAT3 was observed as early as 15–30 min following incubation of MDA-MB-231 cells with 1% (w/v) MH and was maintained for 4–6 h (ref# [23] and Figure 1A). Total STAT3 (t-STAT3) protein levels were not affected by MH (ref# [23] and Figure 1B), which suggest that loss of p-STAT3 is due to post-translational inhibition of tyrosine phosphorylation. Importantly, inhibition of p-STAT3 is paralleled by decreasing levels of gp130 and p-JAK2 proteins (Figure 1C,D), two critical upstream components of the IL-6R signaling pathway [29]. It should be noted that the data in Figure 1A,B is shown here as a control for the new findings shown in Figure 1C,D. 

### 2.2. Constitutive p-STAT3 is Dependent on Autocrine Activation

The presence of constitutively activated p-STAT3 in TNBCs is thought to be due to an autocrine signaling pathway involving IL-6, JAK2, and STAT3 [14]. As demonstrated previously, treatment of MDA-MB-231 cells with 1% MH resulted in a significant inhibition of IL-6 secretion (ref # [23] and Figure 1E), which was observed as early as 1 h after culture initiation in the presence of MH. This was associated with a reduced level of p-STAT3 in the cells, amounting to a ~70% loss in activated STAT3 (Figure 1F). Interestingly, incubation of MDA-MB-231 cells for 4 h in the presence of Brefeldin A, which blocks protein egress from the endoplasmic reticulum, also led to a significant loss of p-STAT3 levels (Figure 1F). This finding highlights the importance of secreted cellular factors, most importantly, IL-6 [30], in maintaining the relatively high levels of constitutively active p-STAT3 in these cells. When cells were cultured in the presence of MH plus Brefeldin A, an almost total loss in p-STAT3 was observed (Figure 1F). 

### 2.3. Exposure to MH Causes a Loss of p-STAT3, gp130, and p-JAK2 in A549 Lung Cancer Cells

**Figure 2:**
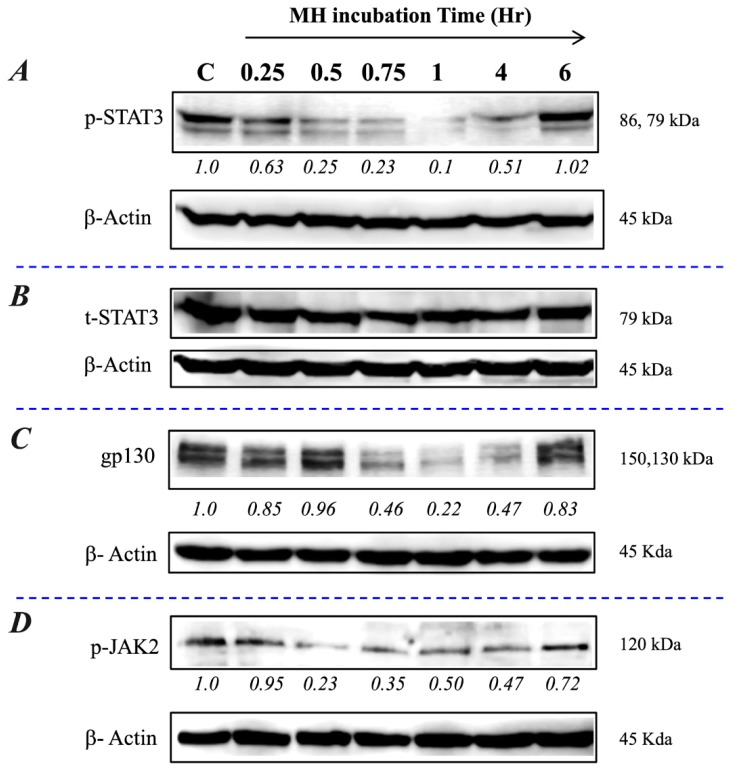
MH inhibits the IL-6 receptor pathway in lung cancer cells. A549 cells were exposed to 1% MH or SC solution and analyzed as described in the Figure 1 legend for MDA-MB-231 cells. Relative levels of p-STAT3 (**A**), total STAT3 (**B**), gp130 (**C**), and p-JAK2 (**D**) were examined by Western blots. The numbers below each blot indicate changes in the band intensity compared to the control, as determined by densitometric analysis. The data are representative of two independent experiments.

We have validated the above findings in an independent lung cancer cell line, A549. This NSCLC cell line is known to express high levels of p-STAT3 constitutively [7]. The data, shown in Figure 2, demonstrate that exposure to 1% MH resulted in a rapid decline in p-STAT3, but not t-STAT3, levels that were first observed at 15 min and was maximal at 1 h (Figure 2A,B). The inhibition of p-STAT3 was accompanied by a decrease in gp130 and p-JAK2 levels (Figure 2C,D), confirming the findings obtained with MDA-MB-231 cells. These data suggest that the inhibitory effect of MH on p-STAT3 levels is not specific to a particular cell line or type of cancer. 

### 2.4. MH Binds Competitively and Specifically to IL-6Rα

**Figure 3:**
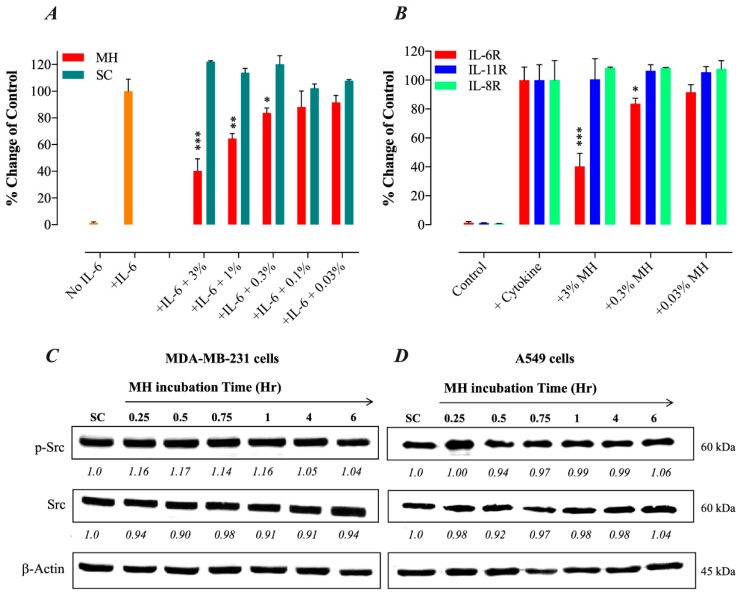
MH inhibits IL-6/STAT3 signaling pathway by selective blockade of IL-6Rα. (**A**) ELISA-based binding assay was performed to test for a possible direct association between MH and IL-6Rα and its consequences on IL-6 binding to its receptor. For the competition assay, various concentrations of MH (0.03–3%) or sugar control solution (SC) were tested for the ability to inhibit the binding of IL-6 protein to immobilized sIL-6Rα competitively. (**B**) A comparison of the ability of MH to bind different cytokine receptors (IL-6Rα, IL-8R, and IL-11Rα). Pre-incubation of various concentrations of MH (3%, 0.3%, and 0.03%) with either recombinant IL-11Rα or IL-8R proteins failed to affect the binding of their respective cytokines. The data depict the % change in the binding of the ligands to their respective receptors in the presence of MH. The data are expressed as means ± SD of 4–6 replicates per group and are pooled from two independent experiments. Asterisks denote statistically significant differences in MH-treated samples compared to control wells (* *p* < 0.05; ** *p* < 0.01; *** *p* < 0.001), as determined by the unpaired, two-tailed Student’s t-test. (**C**,**D**) MH does not alter tyrosine-phosphorylated Src (p-Src) or total Src kinase levels. (**C**) MDA-MB-231 cells were treated with 1% MH for the indicated time points (**C**) and analyzed by Western blotting for the relative protein expression of p-Src, total Src, or β-actin, as control. (**D**) A549 cells were similarly exposed to 1% MH for the indicated times and analyzed for p-Src and total Src expression. The numbers below each blot indicate changes in band intensity compared to control, as determined by densitometric analysis. The data are representative of three independent experiments.

Given the observed inhibitory effects of MH on gp130 protein, we hypothesized that a component(s) of MH could bind directly to the IL-6Rα protein. To test this possibility, we utilized a competitive binding assay using recombinant IL-6Rα and IL-6 reagents and a monoclonal antibody to IL-6. The addition of MH to IL-6Rα-coated wells blocked the binding of its cognate IL-6 ligand in a dose-dependent manner (Figure 3A). Significant inhibition levels of 60%, 36%, and 16% were observed at 3%, 1%, and 0.3% MH concentrations, respectively (Figure 3A). We also studied the possible interaction between MH and two other cytokine receptors with important pro-tumorigenic roles in breast and lung cancer cells, namely IL-11R, which is a member of the IL-6R family [31,32], and the distinct IL-8R (CXCR1) chemokine receptor [33]. Using the above-described competitive binding assay, pre-incubation of MH with either recombinant IL-11Rα or IL-8R proteins failed to affect their binding by their respective cytokines (Figure 3B). Thus, unlike the case with IL-6Rα, MH does not appear to bind to either IL-11Rα or IL-8R in a manner that disrupts the binding of their cognate ligands. To further investigate the selectivity of MH actions in cancer cells, we tested the effect of MH exposure on p-Src and total Src levels in both MDA-MB-231 and A549 cells (Figure 3C,D). The c-Src proto-oncogene is activated by tyrosine phosphorylation in response to multiple growth factors, integrin, and hormone receptors [34]. The data show that MH has no effect on the levels of p-Src or total Src in MDA-MB-231 (Figure 3C) and A549 cells (Figure 3D), suggesting that MH does not interfere with Src-dependent signaling pathways in cancer cells. These findings confirm that MH binds selectively to the IL-6Rα protein with sufficient affinity to compete out the binding of the IL-6 ligand to its receptor. Nevertheless, it is important to note that these findings do not exclude the possibility of MH binding to other receptors expressed by cancer cells.

#### MH Flavonoids Bind to IL-6Rα

**Figure 4:**
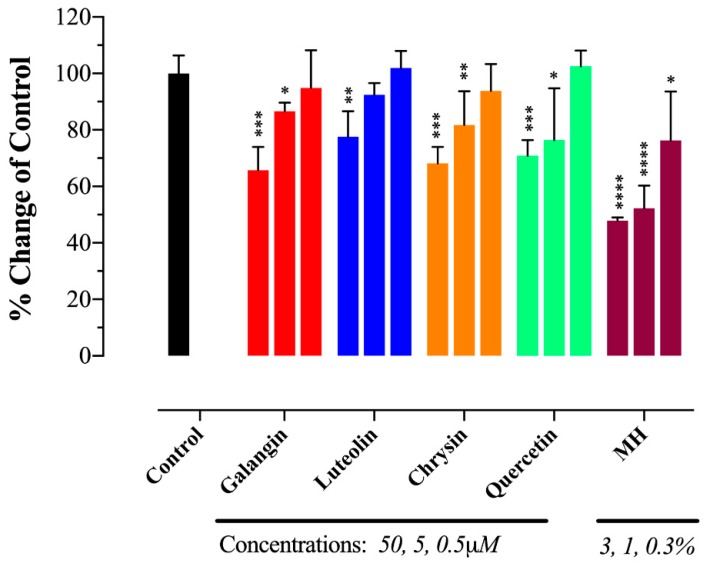
MH flavonoids block IL-6 binding to IL-6Rα. Four major flavonoids in MH, luteolin, chrysin, quercetin, and galangin, were tested individually at the indicated concentrations (0.5, 5, and 50 μM) for binding to IL-6Rα and the ability to compete out the binding of IL-6. The data are expressed as means ± SD of 2–4 replicates per group and are representative of at least two independent experiments. Asterisks denote statistically significant differences in percent inhibition of ligand-receptor binding in MH or flavonoid-treated samples compared to control wells (* *p* < 0.05; ** *p* < 0.01; *** *p* < 0.001; **** *p* < 0.0001), as determined by the unpaired, two-tailed Student’s *t*-test.

We next tested the potential binding of flavonoid compounds to IL-6Rα protein. Four of the major flavonoids present in MH, including luteolin, chrysin, quercetin, and galangin [35], were tested individually for binding to IL-6Rα and their ability to compete out the binding of IL-6. The findings of this analysis, illustrated in Figure 4, show that all four flavonoids could interfere with IL-6 binding to its receptor in a dose-dependent fashion (Figure 4). The extent of the observed inhibition using 50 μM concentration was 34.3%, 22.4%, 31.8%, and 29.2% for galangin, luteolin, chrysin, and quercetin, respectively (Figure 4). At 10-fold lower concentrations, decreased but significant inhibition was still observed with galangin, chrysin and quercetin (Figure 4). It is evident that the degree of inhibition observed with flavonoid compounds at 50 μM concentration was equivalent to the inhibition seen using <1% (~0.3%) MH solution (Figure 4). A fifth flavonoid, pinocembrin, was also tested but no significant binding to IL-6Rα was observed (data not shown). We conclude that at least four of the major MH flavonoids tested have the capacity to associate with, and block ligand binding to, IL-6Rα protein. 

### 2.5. MH Flavonoids Exhibit Differential Inhibitory Capacity on STAT3 Phosphorylation

**Figure 5:**
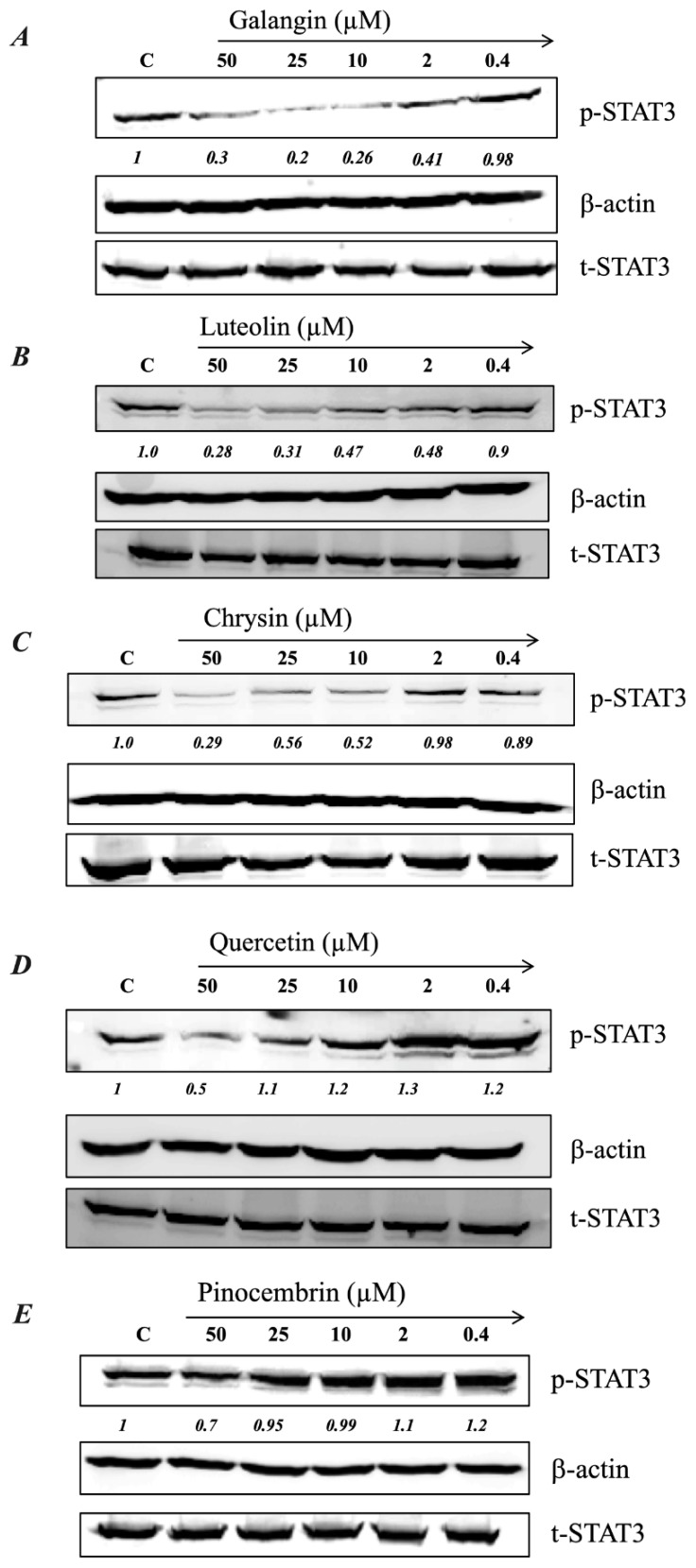
MH flavonoids inhibit p-STAT3 expression in breast cancer cells. Cells were treated for 1 h with various concentrations (0.4–50 μM) of galangin (**A**), luteolin (**B**), chrysin (**C**), quercetin (**D**), and pinocembrin (**E**). Whole-cell extracts were then subjected to Western blotting with antibodies specific to tyrosine-phosphorylated STAT3 (p-STAT3) or total STAT3 (t-STAT3) protein. The numbers below each blot indicate changes in band intensity compared to control, as determined by densitometric analysis. These values were used to calculate the approximate IC_50_ doses (concentrations that induce 50% inhibition in p-STAT3 levels) for each flavonoid, as shown in the corresponding graphs in Figure 6A–E. The data are representative of at least duplicate experiments for each flavonoid compound.

**Figure 6:**
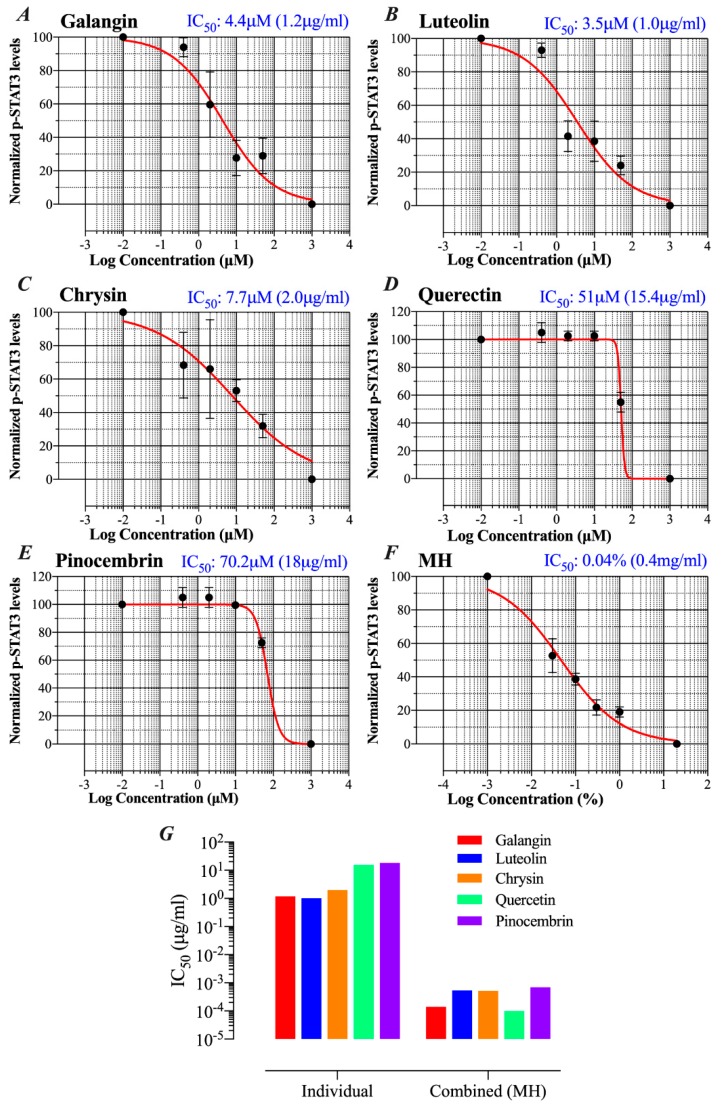
Comparative capacities of flavonoid compounds and MH to inhibit p-STAT3. Following a 1 h-treatment with flavonoids or MH, changes in p-STAT3 levels in breast cancer cells were used to calculate the approximate IC_50_ doses (concentrations that induce 50% inhibition in p-STAT3 levels). Cells were either untreated (control) or treated with various concentrations (0.4–50 μM range) of galangin (**A**), luteolin (**B**), chrysin (**C**), quercetin (**D**), and pinocembrin (**E**). For comparison, the IC_50_ was also estimated for cells treated with MH (0.03%–1% w/v) (F). The IC_50_ determination was done by nonlinear regression best-fit curve analysis (GraphPad Prism) using 10^−2^ μM and 10^3^ μM to represent minimum (untreated control) and maximum doses (theoretical estimate). For MH treatment, the minimum and maximum doses were 10^−3^, and 20% MH, respectively. (**G**) Comparison of IC_50_ values for pSTAT3 inhibition of the different flavonoids when used alone or as part of the whole MH solution. The data are representative of at least duplicate experiments for each flavonoid compound and four experiments for MH.

We showed previously that low concentrations of MH (as little as 0.03% w/v, or 3 mg/mL) could effectively decrease p-STAT3 levels in MDA-MB-231 cells by ~50% [23]. Since flavonoid compounds could demonstrably bind to IL-6Rα, the relative capacity of individual compounds (galangin, luteolin, chrysin, quercetin, and pinocembrin) to inhibit p-STAT3 in MDA-MB-231 cells was investigated next. Representative Western blots are shown in Figure 5 and the estimated concentration of each flavonoid to cause a 50% inhibition (IC_50_) in p-STAT3 levels is illustrated in Figure 6A–E. For comparison, the calculated IC_50_ for MH is also shown (Figure 6F). The findings show that flavonoids exhibit relative differential capacities to inhibit p-STAT3 (Figure 5A–E; Figure 6A–E). Among the tested compounds, luteolin, galangin, and chrysin were the most effective, with an approximate IC_50_ of 3.5, 4.4, and 7.7 μM, respectively (Figure 6A–C). They also exhibited similar dose-responses to that of MH. Quercetin and pinocembrin were the least effective in inhibiting p-STAT3, with approximate IC_50_ of 51 and 70.2 μM, respectively, and exhibited quite distinct dose-response attributes compared to the other flavonoid compounds (Figure 6D,E). We conclude that more than one flavonoid compound could act to suppress the functional activity of p-STAT3 in MDA-MB-231 breast cancer cells, with varying degree of relative efficacy. 

Since the concentration of each flavonoid in MH was previously published [35], we wished to compare the IC_50_ concentration for p-STAT3 inhibition of each flavonoid when used alone or as part of the whole MH solution. The mean reported concentrations of flavonoids in MH are 0.035, 0.136, 0.131, 0.024, and 0.174 mg/100 g MH for galangin, luteolin, chrysin, quercetin, and pinocembrin, respectively [35]. Given that the estimated IC_50_ of MH is 0.04% (~0.4 mg/mL), we could calculate the concentration of each flavonoid in 0.04% solution and compare that with the corresponding concentration estimated to result in 50% inhibition of p-STAT3 when each flavonoid is used in the pure form alone. The results of this comparison highlight an important point, namely that the concentration of each flavonoid compound necessary to induce a 50% inhibition of p-STAT3 activity is 2000 to >160,000-fold higher when used individually compared to when used in combination as MH solution (Figure 6G). We are fully cognizant of the limitations inherent in this rather simplistic comparative analysis, as there are presumably many other constituent compounds within MH that could potentially influence p-STAT3 levels. Nevertheless, this analysis suggests a substantial degree of synergism in the way that flavonoids, with perhaps other bioactive components, act on cancer cells. 

### 2.6. Docking Analysis Reveals Preferential Interaction Between Flavonoid Compounds and IL-6Ra

Despite the fact that IL-6 and IL-11 belong to the same cytokine family, structural differences between human IL-6 and IL-11 in their receptor-binding characteristics have been described [36]. Furthermore, the results of our ELISA binding studies suggest that MH and flavonoids can interact with IL-6Rα but not IL-11Rα proteins. While the crystal structure of IL-6Rα is known [37], that of the IL-11Rα remains undetermined. To further our understanding of the underlying mechanism of MH action and its major flavonoids on the IL-6R signaling pathway, we performed docking analysis of different flavonoid compounds with IL-6Rα chain. The high-resolution crystal structure of the extracellular domains of the human IL-6Rα was used to predict the possible binding interactions of MH flavonoids (luteolin, quercetin, chrysin, galangin, and pinocembrin) to this receptor. We used SwissDock and UCSF Chimera to analyze the interactions of each flavonoid with IL-6Rα protein. The X-ray structure of the extracellular domains of human IL-6Rα consists of the N-terminal Immunoglobulin (Ig) domain (D1, residues 1–93) linked to the classical cytokine binding domains D2 (residues 94–194) and D3 (residues 195–299) [37]. As the cytokine-binding domain has been shown to be responsible for both ligand binding and signal transduction [38], we focused on the docking poses of flavonoids binding in this domain, which is consistent with the available biological data of IL-6Rα receptor. 

**Figure 7:**
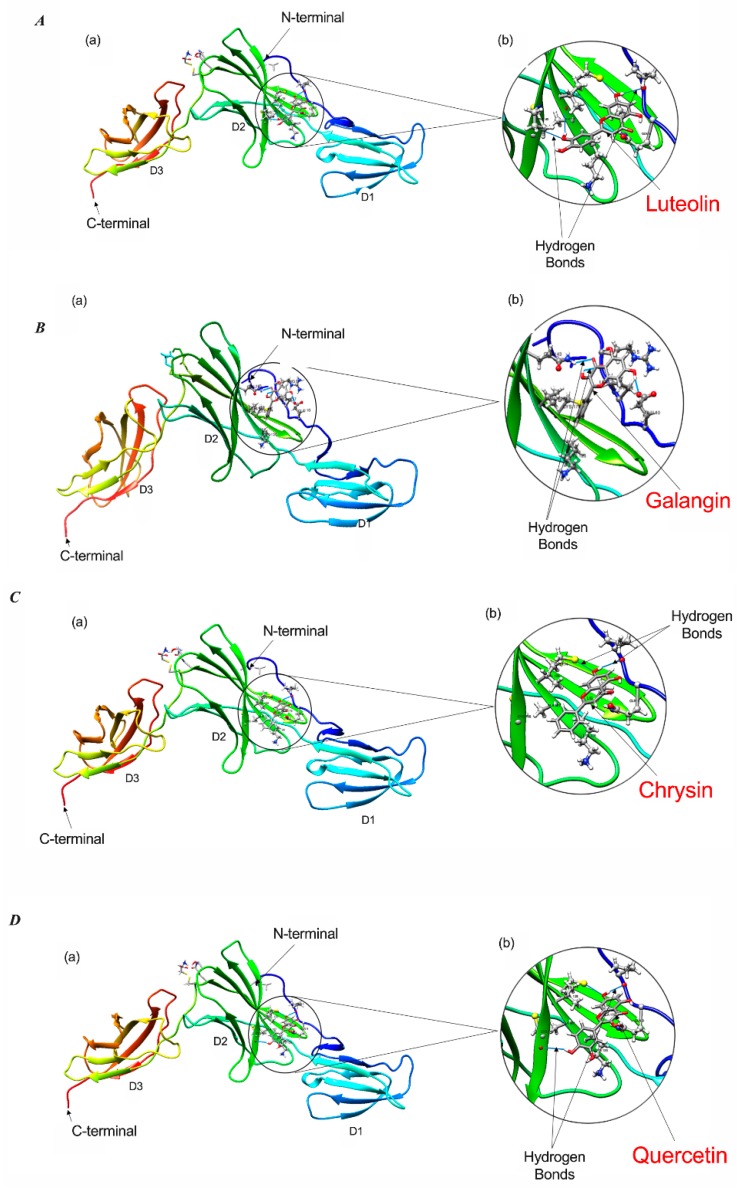

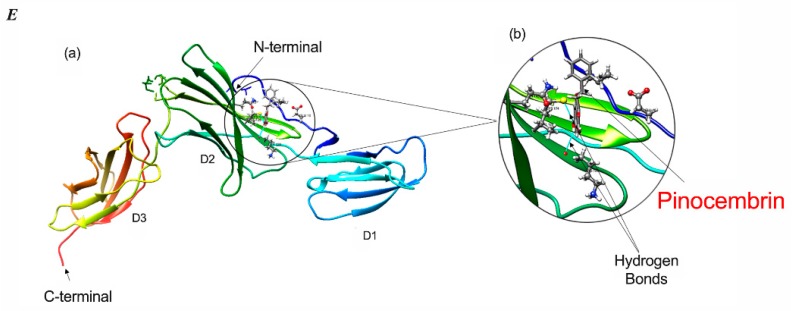
Molecular docking of flavonoid compounds with IL-6Rα protein. Conformational changes of IL-6Rα (PDB ID: 1N26) upon binding with luteolin, quercetin, chrysin, pinocembrin, and galangin (**A**–**E**), respectively. For all figures, sub-figures (**a**) illustrate the docked conformations of flavonoid with each of the interacting amino-acid residues of IL-6Rα protein; Sub-figures (**b**) show enlarged views of the docked conformation of each flavonoid with the residues of IL-6Rα. The β-sheet arrangement and helices (shades of blue, green, and orange for domains D1, D2, and D3, respectively) are shown. The blue lines indicate hydrogen bond formation of flavonoids with surrounding amino-acid residues.

The results of the molecular docking analysis of the binding of flavonoids in the protein pockets of the classical cytokine binding domain D2 of human IL-6Rα are illustrated in Figure 7 and Table 1. The conformation of luteolin shows that it could be able to form four hydrogen bonds in interaction with the residues of Pro 7, Ala127, Cys146, and Cys174, making it a favorable binding site (Figure 7A). The residues Cys146 and Cys174 located on the beta-strands in the NH2-terminal barrel of cytokine-binding domain D2 interacted with the flavonoids. Substitutions in any of the four Cys residues located on such beta strands have been reported to result in a complete loss of ligand binding to IL-6Rα [38]. Hence, the interaction between luteolin and IL-6Rα is likely to inhibit the binding of the IL-6 ligand to the receptor by altering the affinity or protein conformation of the receptor. Similar findings were obtained when binding of the other four flavonoids to IL-6Rα was analyzed (Table 1 and Figure 7B–E). The conformation of quercetin could form three hydrogen bonds with Pro7, Cys146, and Cys174 residues in a single binding pocket of the receptor protein (Figure 7D). In contrast, chrysin docking on D2 domain could be simulated in two different conformations. In the first, chrysin could form two hydrogen bonds with Pro 7 and Cys174 residues (cluster 6; Figure 7C, Table 1). In an alternative conformation, chrysin could form two hydrogen bonds with Cys146 and Ala127 residues of another favorable binding pocket of the receptor (cluster 22; ∆G -6.62; not shown). Pinocembrin could form two hydrogen bonds with Lys 126 and Cys174 in a favorable binding pocket D2 domain, as shown in Figure 7E and Table 1. Finally, the conformation of galangin has three hydrogen bonds with Arg5, Glu10, and Gln 43 in another favorable binding pocket of receptor (Figure 7B, Table 1). Thus, luteolin, quercetin, chrysin, pinocembrin, and galangin flavonoids could interact with domain D2 of IL-6Ra receptor by forming different hydrogen bonds in favorable binding pockets with different binding energies. As reported previously [37], the expected binding position of IL-6 would be in the region of the outer elbow formed at the junction of D2 and D3, characterized by four loops [S106–N110 (L1), K133– P138 (L2), A160–F168 (L3), Q190–G193 (L4)] from D2 and three loops [S227–R233 (L5), M250–H256 (L6), and Q276– Q281 (L7)] from D3. Accordingly, IL-6 would engage residues in some of these loops. In our molecular docking study, the location of binding pockets on the D2 cytokine binding domain is found in the nearby region to the L2 and L3 loops. Therefore, the docking conformations in the present study suggest the binding of flavonoids to IL-6Rα protein can inhibit IL-6 binding by altering the affinities or protein conformation of the receptor. It should be noted that while the in silico docking studies allow us to make predictions on the binding of flavonoids to IL-6Rα, these results require validation by direct flavonoid/IL6-Rα protein interaction studies as well as mutagenesis experiments.

## 3. Discussion

We previously identified p-STAT3 as an early molecular target of MH in cancer. MH causes a rapid loss of p-STAT3 in human breast cancer cells, reducing their proliferation, migration, and invasiveness [23]. In the present study, we demonstrate that exposure to MH leads to a decline in p-STAT3 levels in human A549 lung cancer cells with very similar kinetics to that observed in MDA-MB-231 cells. Moreover, we show that the loss of p-STAT3 is accompanied by a decrease in the levels of gp130 and p-JAK2, two upstream regulators of p-STAT3 activity in breast (MDA-MB-231) and lung (A549) cancer cells. Importantly, our current findings demonstrate, and for the first time, that MH binds directly and competitively to IL-6Rα protein, competing out the binding of IL-6 ligand. To the extent of the limited receptor types tested herein, the binding of MH to IL-6Rα appears to be specific, as no binding to the closely related IL-11Rα or to IL-8R was evident. Moreover, exposure to MH had no effect on the levels of a constitutively active p-Src kinase in MDA-MB-231 and A549 cancer cells. Given that p-Src is induced by different types of growth factors, hormone, and integrin receptors in cancer cells, the lack of its inhibition by MH suggests that the latter does not interfere with ligand binding to multiple receptor types, including PDGF-R, EGF-R, FGF-R, VEGF-R, and IGF-1R [34]. We conclude that the capacity of MH to inhibit p-STAT3 is most likely a consequence of its ability to bind directly to IL-6Rα protein and interfere with the JAK-STAT3 signaling pathway. However, the possibility that MH may also interfere with other pro-tumorigenic receptors in cancer cells cannot be completely excluded.

The IL-6 signaling pathway plays an important role in linking chronic inflammation to tumorigenesis, being directly involved in both cancer initiation and progression [39]. The functional activity of IL-6 is dysregulated in a variety of malignancies, including breast, lung, pancreatic, colorectal, gastric, blood, and skin cancers, and high serum IL-6 levels are associated with bad prognosis in cancer patients [40,41,42]. There are two well-characterized ways in which IL-6R signaling takes place. Classical signaling is initiated by the binding of IL-6 to the membrane-bound IL-6Rα chain, which recruits gp130 to form a hexameric IL-6/IL-6R/gp130 complex, ultimately leading to the phosphorylation and activation of STAT3 [43]. The membrane form of IL-6Rα is expressed only on hepatocytes and some immune cells, such as neutrophils and monocytes/macrophages, and has been suggested to be primarily important for the anti-inflammatory and regenerative activities of IL-6 [44]. The second type of signaling, termed trans-signaling, is dependent on the release of a soluble form of the IL-6Rα (sIL-6Rα) by either alternative splicing of the IL-6Rα transcript or by proteolytic cleavage of the membrane-bound form. Trans-signaling is initiated when a complex is formed between IL-6 and sIL-6Rα, which subsequently binds to the membrane-expressed gp130 unit and triggers downstream events in the pathway [44]. Given that gp130 is ubiquitously expressed in many cell types, including cancer cells, trans-signaling is the predominant form of IL-6 signaling in inflammation and cancer [45,46,47]. Since MDA-MB-231 and A549 cancer cells lack surface expression of IL-6Rα (unpublished data), it is likely that MH exerts its anti-tumor effect via binding to sIL-6Rα, inhibiting its association with the IL-6 cytokine and ultimately preventing the triggering of the IL-6 trans-signaling pathway.

There is mounting evidence showing that blocking of IL-6 trans-signaling not only inhibits tumor initiation in animal models but can also interfere with the growth of established tumors [45,46,48,49]. An inhibitory mAb, Tocilizumab that binds human IL-6R and blocks its binding to IL-6 has been developed and is currently approved for the treatment of autoimmune conditions [50]. Moreover, several studies have highlighted the potential of using inhibitors of IL-6R in preclinical cancer models [51,52,53,54]. The current study is the first to demonstrate the ability of natural compounds, such as MH, to bind sIL-6R and interfere with IL-6 trans-signaling in cancer cells.

The potential contribution of flavonoid compounds to the binding of the IL-6Rα protein was also investigated. The data revealed that with the exception of pinocembrin, each of the other major flavonoids in MH (luteolin, quercetin, chrysin, and galangin) is able to bind IL-6Rα and, in the 5–50 μM concentration range, resulting in a moderate, albeit statistically significant, inhibition (30–35%) in the binding of the cognate IL-6 ligand. Molecular docking studies confirmed that, due to the similarity in their structure, each flavonoid compound could bind IL-6Rα at a site that would be predicted to affect ligand binding. Functional studies demonstrated that flavonoid compounds also have a differential capacity to inhibit p-STAT3. In terms of relative IC_50_, luteolin, galangin and chrysin were most effective in inhibiting p-STAT3 (IC_50_ of 3.5 μM, 4.4 μM, and 7.7 μM, respectively), while quercetin and pinocembrin were the least effective (IC_50_ of 51 μM and 70.2 μM, respectively). The fact that pinocembrin exhibited the lowest efficacy in p-STAT3 inhibition correlates well with our inability to detect any binding between this flavonoid and IL-6Rα at the maximum dose used (50 μM). It is important to state that the IC_50_ estimates based on densitometry quantification of p-STAT3 levels by Western blots are only semi-quantitative. As such, our data serve to solely illustrate the relative potency of the different flavonoids in p-STAT3 inhibition. Taken together, these findings suggest flavonoid compounds may well be responsible for MH-mediated inhibition of the IL-6/STAT3 signaling pathway in human breast and lung cancer cells.

We undertook a comparative analysis of the IC_50_ for p-STAT3 inhibition of each flavonoid compound when used alone or as part of the MH mixture. This analysis revealed that much higher concentrations of each major flavonoid are needed to affect p-STAT3 levels in breast cancer cells when used in pure form in comparison with the whole MH solution. In fact, based on the known concentration of each flavonoid compound in MH, we estimate that MH as a mixture is 2000 to 160,000-fold more efficacious in blocking STAT3 activity than any flavonoid used individually. These findings are in line with previously published data by other investigators showing that a combination of polyphenols or mixtures of polyphenols with vitamins, amino acids, and other micronutrients exhibited superior anti-cancer activity than individual compounds [55].

The capacity of some flavonoids to inhibit p-STAT3 activity has been described. A high dose of quercetin (66 μM) was found to reduce p-STAT3 levels in lung cancer cells after a long incubation period (12–24 h) by an indirect effect on NF-κB activation and IL-6 production [56]. Similarly, high concentrations (40–80 μM) of chrysin were recently shown to inhibit p-STAT3 via the production of ROS in human bladder cancer cells [57]. This was observed mostly after 24 h of incubation with chrysin, suggesting an indirect effect. Of note, no evidence for ROS generation was observed in MH-treated MDA-MB-231 human breast cancer cells [23], indicating that inhibition of p-STAT3 in these cells is largely ROS-independent. Furthermore, several studies reported the capacity of luteolin to inhibit STAT3 in different types of cancer cells, including breast, stomach, lung, liver, pancreas, cervix, and bile duct cancers [58,59,60,61,62,63,64]. Mechanistically, luteolin appears to directly bind to Hsp90, a molecular chaperone that stabilizes p-STAT3 [59]. This, in turn, inhibits Hsp90 and promotes the degradation of p-STAT3 [58,59]. Whether a similar mechanism could underlie the inhibition observed in MH-treated breast cancer cells is unknown. It is interesting to note; however, that luteolin-mediated inhibitory effects on different cancer cell types were observed at 20–50 μM concentration range (~6–14 μg/mL), which are at least 400–1000-fold higher than the concentration of luteolin in 1% (w/v) MH solution used in the present study [23,35]. Given the above findings with the different flavonoids, it is not unreasonable to propose that flavonoids could exert their effects through interfering with IL-6R signaling.

In addition to IL-6, breast and lung cancer cells are known to rely on autocrine signaling by IL-11 for their tumor-associated functions, including survival, invasion, and metastasis [31,32]. IL-6 and IL-11 cytokines are closely related and signal exclusively through the signal-transducing receptor gp130 to activate the JAK-STAT3 pathway [65]. Moreover, available evidence indicates that both IL-6 and IL-11 cytokines are produced constitutively at equivalent levels by breast cancer cells [66,67]. Our findings suggest that MH interacts selectively with IL-6Rα and interferes with its ligand binding. Nevertheless, in our model system, exposure of breast cancer cells to MH leads to a rapid loss of >80% of activated p-STAT3 protein [23], which is also associated with decreased gp130 and p-JAK2 levels. The fact that the substantial loss of p-STAT3 is seen under conditions affecting only IL-6/IL-6R signaling suggests that the contribution of IL-11/IL-11R to maintaining constitutive p-STAT3 levels in these cancer cells is relatively small. Alternatively, these findings may suggest that MH components could act at additional levels downstream of the IL-6R signaling pathway. A case in point is the previous demonstration that luteolin could also interact with Hsp90 and promote the degradation of p-STAT3 [59]. Therefore, studies in which the effect of MH or its flavonoid components is tested on cancer cells in the presence of exogenous IL-6 or IL-11 could further our understanding of the molecular targets within breast and lung cancer cells.

In conclusion, the present findings identify IL-6Rα as a selective target for MH and its flavonoid constituents. This is based on three lines of evidence. First, MH binds to and interferes with the ligand binding to IL-6Rα, but not the closely related IL-11Rα or IL-8R proteins. Second, in silico docking studies reveal favorable binding to IL-6Rα at sites predicted to affect ligand binding. Third, MH does not inhibit tyrosine phosphorylation of c-Src kinase, another major proto-oncogene in a variety of human cancers. Since p-Src is induced by a large group of growth factor, integrin and hormonal receptors [34], the absence of any inhibition by MH suggests a lack of association with these other receptors/signaling pathways. Identification of the molecular targets of MH and characterization of the mechanisms underlying its inhibitory effects on cancer cells will lead to a better understanding of the applicability of, and the most appropriate approach for the use of, MH and/or its bioactive constituents as therapeutic agents in cancer treatment [68]. 

## 4. Materials and Methods

### 4.1. Cell Line and Reagents

The human breast cancer cell line MDA-MB-231 was maintained in complete DMEM supplemented with 10% fetal bovine serum (FBS) (Hyclone-GE Healthcare life Sciences, Pittsburg, USA), as previously described [22]. The human NSCLC cell line A549 was maintained in RPMI 1640 (Hyclone) supplemented with antibiotics (penicillin 50 U/mL; streptomycin 50 µg/mL) and 10% FBS. In all experiments, cell viability was higher than 99% using trypan blue dye exclusion. MH (UMF^®^ 20**+**) was purchased from ApiHealth (Auckland, New Zealand). As a control for MH, we used a sugar solution (designated Sugar Control or SC) containing equivalent concentrations of the three major sugars in honey (38.2% fructose, 31.3% Glucose, and 1.3% Sucrose), as described [23]. The MH flavonoid compounds (luteolin, quercetin, galangin, chrysin, and pinocembrin) were purchased from Sigma (St. Louis, MO, USA). For all reagents, appropriate dilutions to the desired concentrations were freshly made before addition to the cell cultures. Flavonoid stocks were made in DMSO and diluted to required concentrations in 5% DMEM for cell culture or in PBS for use in ELISA binding assay. Similarly-diluted DMSO solution in 5% DMEM or PBS was used as a control for the flavonoids. 

### 4.2. Western Blot Analysis

The expression of different proteins involved in IL-6 signaling pathway was analyzed by Western blots, as previously described [23,69]. Briefly, MDA-MB-231 or A549 cells (2 × 10^6^ cells/well) were seeded overnight in DMEM/RPMI plus 2% FBS, followed by incubation with MH or sugar control (SC) in 5% FBS-DMEM/RPMI for different times, as indicated in the figures. In some studies, we investigated the effect of treatment with different flavonoid compounds on p-STAT3 expression using a range of doses (0.4–50 μM). In other experiments, we also studied the effect of incubating MDA-MB-231 cells in the presence of Brefeldin A (BFA; 1 mg/mL) alone or with 1% MH for 4 h on p-STAT3 levels. Cell extracts were prepared in RIPA buffer and subjected to Western blot analysis using antibodies (purchased from Cell Signaling Technology, Danvers, MA, USA) specific to total STAT3 (t-STAT3), tyrosine-phosphorylated (Tyr705) STAT3 (p-STAT3), gp130, JAK2, tyrosine-phosphorylated (Tyr1007/1008) JAK2 (p-JAK2), tyrosine-phosphorylated (Tyr416) Src family (p-Src), total Src, and β-actin. Densitometric analysis of the band intensity on the blots was done using ImageJ (National Institutes of Health, USA). We modeled the binding inhibition pharmacodynamics of p-STAT3 levels as standard drug-receptor interactions based on mass-action kinetics. Specifically, dose response was modelled as:

$$Y=\frac{\left[L\right]}{\left[L\right]+K}\;$$

where *Y* is the receptor occupancy, *L* is the ligand concentration, and *K* is a compound-specific association constant.

### 4.3. Enzyme-Linked Immunosorbent Assay

The effect of MH treatment on IL-6 secretion was analyzed by ELISA, following established protocols [23,70]. MDA-MB-231 cells were seeded overnight in 2% FBS-DMEM in six-well plates (2 × 10^6^/well), and then incubated in 5% FBS medium in the presence of 1% MH for different times (0.5–4 h). Cell-free culture supernatants were then collected and analyzed for IL-6 content by a specific ELISA (Biolegend, San Diego, CA, USA) according to the manufacturer’s recommendations. 

### 4.4. Competitive Cytokine Receptor Binding Assays

We used an ELISA-based assay [71] to determine if MH can bind to recombinant IL-6Rα, IL-8R, and IL-11Rα proteins (all purchased from R&D Systems; Minneapolis, MN, USA). IL-6Rα (2 μg/mL), IL-8R (0.5 μg/mL), or IL-11Rα (1 μg/mL) proteins were coated on 96-well plates by overnight incubation at 4 °C. The plates were then washed with PBS + 0.05% tween 20 and blocked with PBS containing 1% BSA for 1 h at room temperature. For competition assay, various concentrations of MH (0.03–3%) were added to recombinant receptor-coated plates and incubated for 1 h at room temperature. After thorough washing, recombinant IL-6, IL-8, or IL-11 cytokines were added (50 ng/mL) and incubated for 1 h at 37 °C. The plates were then thoroughly washed and biotin-conjugated mAbs to IL-6 (0.25 µg/mL), IL-8 (0.5 µg/mL) or IL-11 (0.5 µg/mL) were added for 1 h at room temperature. After another round of washing, HRP-conjugated streptavidin was added for 30 min, washed, and developed by adding 3,3′,5,5′-Tetramethylbenzidine (TMB) substrate for 10 min. Additionally, the potential effect of flavonoid compounds (luteolin, quercetin, galangin, and chrysin; dose range 0.5–50 μM) on binding to IL-6Rα was investigated in the same assay. The dose-response was modeled based on mass-action kinetic drug-response interactions.

### 4.5. Docking Analysis

The high-resolution crystal structure of the extracellular domains of the human interleukin-6 receptor alpha-chain [37] (IL-6Rα, PDB code: 1N26) from the RCSB protein data bank (https://www.rcsb.org/structure/1N26) was fetched in UCSF Chimera [72] to retain the protein structure. The protein structure of IL-6Rα was used to predict the possible binding interactions of each flavonoid (luteolin, quercetin, chrysin, galangin, and pinocembrin) with it. The flavonoids were fetched by their PubChem ID into the UCSF Chimera, and their MOL2 files with all hydrogens and 3D coordinates were submitted to the web-based SwissDock program along with the PDB files of IL-6Rα structure for docking study. The docking was performed at default parameters [73]. The predicted docking files generated from Swiss dock were visualized and analyzed in UCSF Chimera to study interactions of each flavonoid with the IL-6Rα protein. Our approach to select a docking pose combined the docking characteristics between the IL-6Rα and the structurally similar flavonoid ligands, identification of experimentally reported mutated residues that inhibit ligand binding to the receptor, hydrogen bonds, the domain reported for the cytokine binding to the receptor and ligand-receptor binding energies. 

## 5. Statistical Analysis

Statistical significance between control and treated groups was analyzed using the unpaired, two-tailed Student’s *t*-test, using the statistical program of GraphPad Prism version 6 software (San Diego, CA, USA). For multiple comparisons, we used ordinary two-way ANOVA (GraphPad Prism). Differences between the experimental groups were considered significant when *p* values were <0.05. Nonlinear regression best-fit curve analysis was used to determine the IC_50_ for p-STAT3 inhibition. 

## Figures and Tables

**Table 1:** Docking characteristics between the major MH flavonoids and IL-6Rα protein, highlighting the hydrogen bonds formed between each flavonoid and the residues of IL-6Rα.

Flavonoid	Cluster	Estimated ∆G (kcal/mol)	Hydrogen Bonds
Luteolin (Lut)	5	−6.72	Lut H7−−Pro7 O (1.851 Å) Lut H9−−Ala127 O (2.100 Å) Cys146 HN−−Lut O5 (2.341 Å) Cys174 SG−−Lut O3 (3.205 Å)
Quercetin (Que)	8	−6.79	Que H6−−Pro7 O (2.323 Å) Que H10−−Cys146 O (2.437 Å) Cys174 SG−−Que O3 (3.495 Å)
Chrysin (Chr)	6	−6.42	Chr H9−−Pro7 O (2.072 Å) Cys174 SG−−Chr O3 (3.307 Å)
Pinocembrin (Pin)	26	−6.25	Pin H11−−Lys126 O (2. 396 Å) Cys174SG−−Pin O3 (3.472Å)
Galangin (Gal)	14	−6.83	Gal H10−−Arg5 O (2.572 Å) Gal H8−−Glu10 OE2 (1.983 Å) Gln143 HE21−−Gal O1 (2.651 Å)

## Abbreviations

MH	Manuka honey
TNBC	Triple negative breast cancer
NSCLC	Non-small cell lung cancer
IL-6	Interleukin-6
IL-6Rα	IL-6 receptor α chain
IL-8R	IL-8 receptor
IL-11Rα	IL-11 receptor α chain
STAT3	signal transducer and activator of transcription 3
p-STAT3	tyrosine-phosphorylated STAT3
gp130	Glycoprotein 130
JAK1/2	Janus kinase 1/2
p-JAK2	tyrosine-phosphorylated JAK2
SC	Sugar control

## References

[1] Hanahan D., Weinberg R.A. (2011). Hallmarks of cancer: The next generation. Cell.

[2] Santa-Maria C.A., Gradishar W.J. (2015). Changing Treatment Paradigms in Metastatic Breast Cancer: Lessons Learned. JAMA Oncol..

[3] Foulkes W.D., Smith I.E., Reis-Filho J.S. (2010). Triple-negative breast cancer. N. Engl. J. Med..

[4] Zappa C., Mousa S.A. (2016). Non-small cell lung cancer: Current treatment and future advances. Transl. Lung Cancer Res..

[5] Knupfer H., Preiss R. (2007). Significance of interleukin-6 (IL-6) in breast cancer (review). Breast Cancer Res. Treat..

[6] Corvinus F.M., Orth C., Moriggl R., Tsareva S.A., Wagner S., Pfitzner E.B., Baus D., Kaufmann R., Huber L.A., Zatloukal K. (2005). Persistent STAT3 activation in colon cancer is associated with enhanced cell proliferation and tumor growth. Neoplasia.

[7] Song L., Turkson J., Karras J.G., Jove R., Haura E.B. (2003). Activation of Stat3 by receptor tyrosine kinases and cytokines regulates survival in human non-small cell carcinoma cells. Oncogene.

[8] Taga T., Kishimoto T. (1997). Gp130 and the interleukin-6 family of cytokines. Annu. Rev. Immunol..

[9] Naugler W.E., Karin M. (2008). The wolf in sheep’s clothing: The role of interleukin-6 in immunity, inflammation and cancer. Trends Mol. Med..

[10] Chiu J.J., Sgagias M.K., Cowan K.H. (1996). Interleukin 6 acts as a paracrine growth factor in human mammary carcinoma cell lines. Clin. Cancer Res..

[11] Conze D., Weiss L., Regen P.S., Bhushan A., Weaver D., Johnson P., Rincon M. (2001). Autocrine production of interleukin 6 causes multidrug resistance in breast cancer cells. Cancer Res..

[12] Berishaj M., Gao S.P., Ahmed S., Leslie K., Al-Ahmadie H., Gerald W.L., Bornmann W., Bromberg J.F. (2007). Stat3 is tyrosine-phosphorylated through the interleukin-6/glycoprotein 130/Janus kinase pathway in breast cancer. Breast Cancer Res..

[13] Sullivan N.J., Sasser A.K., Axel A.E., Vesuna F., Raman V., Ramirez N., Oberyszyn T.M., Hall B.M. (2009). Interleukin-6 induces an epithelial-mesenchymal transition phenotype in human breast cancer cells. Oncogene.

[14] Marotta L.L., Almendro V., Marusyk A., Shipitsin M., Schemme J., Walker S.R., Bloushtain-Qimron N., Kim J.J., Choudhury S.A., Maruyama R. (2011). The JAK2/STAT3 signaling pathway is required for growth of CD44(+)CD24(-) stem cell-like breast cancer cells in human tumors. J. Clin. Investig..

[15] Chang Q., Bournazou E., Sansone P., Berishaj M., Gao S.P., Daly L., Wels J., Theilen T., Granitto S., Zhang X. (2013). The IL-6/JAK/Stat3 feed-forward loop drives tumorigenesis and metastasis. Neoplasia.

[16] Yanagawa H., Sone S., Takahashi Y., Haku T., Yano S., Shinohara T., Ogura T. (1995). Serum levels of interleukin 6 in patients with lung cancer. Br. J. Cancer.

[17] Yeh H.H., Lai W.W., Chen H.H., Liu H.S., Su W.C. (2006). Autocrine IL-6-induced Stat3 activation contributes to the pathogenesis of lung adenocarcinoma and malignant pleural effusion. Oncogene.

[18] Haura E.B., Livingston S., Coppola D. (2006). Autocrine interleukin-6/interleukin-6 receptor stimulation in non-small-cell lung cancer. Clin. Lung Cancer.

[19] Banerjee K., Resat H. (2016). Constitutive activation of STAT3 in breast cancer cells: A review. Int. J. Cancer.

[20] Harada D., Takigawa N., Kiura K. (2014). The Role of STAT3 in Non-Small Cell Lung Cancer. Cancers (Basel).

[21] Rokavec M., Oner M.G., Li H., Jackstadt R., Jiang L., Lodygin D., Kaller M., Horst D., Ziegler P.K., Schwitalla S. (2014). IL-6R/STAT3/miR-34a feedback loop promotes EMT-mediated colorectal cancer invasion and metastasis. J. Clin. Investig..

[22] Fernandez-Cabezudo M.J., El-Kharrag R., Torab F., Bashir G., George J.A., El-Taji H., al-Ramadi B.K. (2013). Intravenous administration of manuka honey inhibits tumor growth and improves host survival when used in combination with chemotherapy in a melanoma mouse model. PLoS ONE.

[23] Aryappalli P., Al-Qubaisi S.S., Attoub S., George J.A., Arafat K., Ramadi K.B., Mohamed Y.A., Al-Dhaheri M.M., Al-Sbiei A., Fernandez-Cabezudo M.J. (2017). The IL-6/STAT3 Signaling Pathway Is an Early Target of Manuka Honey-Induced Suppression of Human Breast Cancer Cells. Front. Oncol..

[24] Bromann P.A., Korkaya H., Courtneidge S.A. (2004). The interplay between Src family kinases and receptor tyrosine kinases. Oncogene.

[25] Zhang S., Huang W.C., Li P., Guo H., Poh S.B., Brady S.W., Xiong Y., Tseng L.M., Li S.H., Ding Z. (2011). Combating trastuzumab resistance by targeting SRC, a common node downstream of multiple resistance pathways. Nat. Med..

[26] Playford M.P., Schaller M.D. (2004). The interplay between Src and integrins in normal and tumor biology. Oncogene.

[27] Zhang S., Huang W.C., Zhang L., Zhang C., Lowery F.J., Ding Z., Guo H., Wang H., Huang S., Sahin A.A. (2013). SRC family kinases as novel therapeutic targets to treat breast cancer brain metastases. Cancer Res..

[28] Silva C.M. (2004). Role of STATs as downstream signal transducers in Src family kinase-mediated tumorigenesis. Oncogene.

[29] Huynh J., Etemadi N., Hollande F., Ernst M., Buchert M. (2017). The JAK/STAT3 axis: A comprehensive drug target for solid malignancies. Semin. Cancer Biol..

[30] Hartman Z.C., Poage G.M., den Hollander P., Tsimelzon A., Hill J., Panupinthu N., Zhang Y., Mazumdar A., Hilsenbeck S.G., Mills G.B. (2013). Growth of triple-negative breast cancer cells relies upon coordinate autocrine expression of the proinflammatory cytokines IL-6 and IL-8. Cancer Res..

[31] Johnstone C.N., Chand A., Putoczki T.L., Ernst M. (2015). Emerging roles for IL-11 signaling in cancer development and progression: Focus on breast cancer. Cytokine Growth Factor. Rev..

[32] Zhao M., Liu Y., Liu R., Qi J., Hou Y., Chang J., Ren L. (2018). Upregulation of IL-11, an IL-6 Family Cytokine, Promotes Tumor Progression and Correlates with Poor Prognosis in Non-Small Cell Lung Cancer. Cell. Physiol. Biochem..

[33] Waugh D.J., Wilson C. (2008). The interleukin-8 pathway in cancer. Clin. Cancer Res..

[34] Parsons S.J., Parsons J.T. (2004). Src family kinases, key regulators of signal transduction. Oncogene.

[35] Chan C.W., Deadman B.J., Manley-Harris M., Wilkins A.L., Alber D.G., Harry E. (2013). Analysis of the flavonoid component of bioactive New Zealand manuka (*Leptospermum scoparium*) honey and the isolation, characterisation and synthesis of an unusual pyrrole. Food Chem..

[36] Putoczki T.L., Dobson R.C., Griffin M.D. (2014). The structure of human interleukin-11 reveals receptor-binding site features and structural differences from interleukin-6. Acta Crystallogr. D Biol. Crystallogr..

[37] Varghese J.N., Moritz R.L., Lou M.Z., Van Donkelaar A., Ji H., Ivancic N., Branson K.M., Hall N.E., Simpson R.J. (2002). Structure of the extracellular domains of the human interleukin-6 receptor alpha -chain. Proc. Natl. Acad. Sci. USA.

[38] Yawata H., Yasukawa K., Natsuka S., Murakami M., Yamasaki K., Hibi M., Taga T., Kishimoto T. (1993). Structure-function analysis of human IL-6 receptor: Dissociation of amino acid residues required for IL-6-binding and for IL-6 signal transduction through gp130. EMBO J..

[39] Taniguchi K., Karin M. (2014). IL-6 and related cytokines as the critical lynchpins between inflammation and cancer. Semin. Immunol..

[40] Salgado R., Junius S., Benoy I., Van Dam P., Vermeulen P., Van Marck E., Huget P., Dirix L.Y. (2003). Circulating interleukin-6 predicts survival in patients with metastatic breast cancer. Int. J. Cancer.

[41] Heikkila K., Ebrahim S., Lawlor D.A. (2008). Systematic review of the association between circulating interleukin-6 (IL-6) and cancer. Eur. J. Cancer.

[42] Lippitz B.E. (2013). Cytokine patterns in patients with cancer: A systematic review. Lancet Oncol..

[43] Boulanger M.J., Chow D.C., Brevnova E.E., Garcia K.C. (2003). Hexameric structure and assembly of the interleukin-6/IL-6 alpha-receptor/gp130 complex. Science.

[44] Rose-John S. (2012). IL-6 trans-signaling via the soluble IL-6 receptor: Importance for the pro-inflammatory activities of IL-6. Int. J. Biol. Sci..

[45] Becker C., Fantini M.C., Schramm C., Lehr H.A., Wirtz S., Nikolaev A., Burg J., Strand S., Kiesslich R., Huber S. (2004). TGF-beta suppresses tumor progression in colon cancer by inhibition of IL-6 trans-signaling. Immunity.

[46] Lesina M., Kurkowski M.U., Ludes K., Rose-John S., Treiber M., Kloppel G., Yoshimura A., Reindl W., Sipos B., Akira S. (2011). Stat3/Socs3 activation by IL-6 transsignaling promotes progression of pancreatic intraepithelial neoplasia and development of pancreatic cancer. Cancer Cell.

[47] Scheller J., Garbers C., Rose-John S. (2014). Interleukin-6: From basic biology to selective blockade of pro-inflammatory activities. Semin. Immunol..

[48] Fisher D.T., Chen Q., Skitzki J.J., Muhitch J.B., Zhou L., Appenheimer M.M., Vardam T.D., Weis E.L., Passanese J., Wang W.C. (2011). IL-6 trans-signaling licenses mouse and human tumor microvascular gateways for trafficking of cytotoxic T cells. J. Clin. Investig..

[49] Grivennikov S., Karin E., Terzic J., Mucida D., Yu G.Y., Vallabhapurapu S., Scheller J., Rose-John S., Cheroutre H., Eckmann L. (2009). IL-6 and Stat3 are required for survival of intestinal epithelial cells and development of colitis-associated cancer. Cancer Cell.

[50] Kang S., Tanaka T., Narazaki M., Kishimoto T. (2019). Targeting Interleukin-6 Signaling in Clinic. Immunity.

[51] Oguro T., Ishibashi K., Sugino T., Hashimoto K., Tomita S., Takahashi N., Yanagida T., Haga N., Aikawa K., Suzutani T. (2013). Humanised antihuman IL-6R antibody with interferon inhibits renal cell carcinoma cell growth in vitro and in vivo through suppressed SOCS3 expression. Eur. J. Cancer.

[52] Ando K., Takahashi F., Motojima S., Nakashima K., Kaneko N., Hoshi K., Takahashi K. (2013). Possible role for tocilizumab, an anti-interleukin-6 receptor antibody, in treating cancer cachexia. J. Clin. Oncol..

[53] Nagasaki T., Hara M., Nakanishi H., Takahashi H., Sato M., Takeyama H. (2014). Interleukin-6 released by colon cancer-associated fibroblasts is critical for tumour angiogenesis: Anti-interleukin-6 receptor antibody suppressed angiogenesis and inhibited tumour-stroma interaction. Br. J. Cancer.

[54] Wakabayashi H., Hamaguchi T., Nagao N., Kato S., Iino T., Nakamura T., Sudo A. (2018). Interleukin-6 receptor inhibitor suppresses bone metastases in a breast cancer cell line. Breast Cancer.

[55] Niedzwiecki A., Roomi M.W., Kalinovsky T., Rath M. (2016). Anticancer Efficacy of Polyphenols and Their Combinations. Nutrients.

[56] Mukherjee A., Khuda-Bukhsh A.R. (2015). Quercetin Down-regulates IL-6/STAT-3 Signals to Induce Mitochondrial-mediated Apoptosis in a Nonsmall- cell Lung-cancer Cell Line, A549. J. Pharmacopunct..

[57] Xu Y., Tong Y., Ying J., Lei Z., Wan L., Zhu X., Ye F., Mao P., Wu X., Pan R. (2018). Chrysin induces cell growth arrest, apoptosis, and ER stress and inhibits the activation of STAT3 through the generation of ROS in bladder cancer cells. Oncol. Lett..

[58] Selvendiran K., Koga H., Ueno T., Yoshida T., Maeyama M., Torimura T., Yano H., Kojiro M., Sata M. (2006). Luteolin promotes degradation in signal transducer and activator of transcription 3 in human hepatoma cells: An implication for the antitumor potential of flavonoids. Cancer Res..

[59] Fu J., Chen D., Zhao B., Zhao Z., Zhou J., Xu Y., Xin Y., Liu C., Luo L., Yin Z. (2012). Luteolin induces carcinoma cell apoptosis through binding Hsp90 to suppress constitutive activation of STAT3. PLoS ONE.

[60] Yang M.Y., Wang C.J., Chen N.F., Ho W.H., Lu F.J., Tseng T.H. (2014). Luteolin enhances paclitaxel-induced apoptosis in human breast cancer MDA-MB-231 cells by blocking STAT3. Chem. Biol. Interact..

[61] Aneknan P., Kukongviriyapan V., Prawan A., Kongpetch S., Sripa B., Senggunprai L. (2014). Luteolin arrests cell cycling, induces apoptosis and inhibits the JAK/STAT3 pathway in human cholangiocarcinoma cells. Asian Pac. J. Cancer Prev..

[62] Huang X., Dai S., Dai J., Xiao Y., Bai Y., Chen B., Zhou M. (2015). Luteolin decreases invasiveness, deactivates STAT3 signaling, and reverses interleukin-6 induced epithelial-mesenchymal transition and matrix metalloproteinase secretion of pancreatic cancer cells. Oncol. Targets Ther..

[63] Song S., Su Z., Xu H., Niu M., Chen X., Min H., Zhang B., Sun G., Xie S., Wang H. (2017). Luteolin selectively kills STAT3 highly activated gastric cancer cells through enhancing the binding of STAT3 to SHP-1. Cell Death Dis..

[64] Sonoki H., Tanimae A., Endo S., Matsunaga T., Furuta T., Ichihara K., Ikari A. (2017). Kaempherol and Luteolin Decrease Claudin-2 Expression Mediated by Inhibition of STAT3 in Lung Adenocarcinoma A549 Cells. Nutrients.

[65] Matadeen R., Hon W.C., Heath J.K., Jones E.Y., Fuller S. (2007). The dynamics of signal triggering in a gp130-receptor complex. Structure.

[66] Crichton M.B., Nichols J.E., Zhao Y., Bulun S.E., Simpson E.R. (1996). Expression of transcripts of interleukin-6 and related cytokines by human breast tumors, breast cancer cells, and adipose stromal cells. Mol. Cell Endocrinol..

[67] Lacroix M., Siwek B., Marie P.J., Body J.J. (1998). Production and regulation of interleukin-11 by breast cancer cells. Cancer Lett..

[68] Afrin S., Haneefa S.M., Fernandez-Cabezudo M.J., Giampieri F., al-Ramadi B.K., Battino M. (2019). Therapeutic and preventive properties of honey and its major bioactive compounds in cancer: An evidence-based review. Nutr. Res. Rev..

[69] Al-Ramadi B.K., Zhang H., Bothwell A.L. (1998). Cell-cycle arrest and apoptosis hypersusceptibility as a consequence of Lck deficiency in nontransformed T lymphocytes. Proc. Natl. Acad. Sci. USA.

[70] Fernandez-Cabezudo M.J., Lorke D.E., Azimullah S., Mechkarska M., Hasan M.Y., Petroianu G.A., al-Ramadi B.K. (2010). Cholinergic stimulation of the immune system protects against lethal infection by Salmonella enterica serovar Typhimurium. Immunology.

[71] Su J.L., Lai K.P., Chen C.A., Yang C.Y., Chen P.S., Chang C.C., Chou C.H., Hu C.L., Kuo M.L., Hsieh C.Y. (2005). A novel peptide specifically binding to interleukin-6 receptor (gp80) inhibits angiogenesis and tumor growth. Cancer Res..

[72] Pettersen E.F., Goddard T.D., Huang C.C., Couch G.S., Greenblatt D.M., Meng E.C., Ferrin T.E. (2004). UCSF Chimera—A visualization system for exploratory research and analysis. J. Comput. Chem..

[73] Grosdidier A., Zoete V., Michielin O. (2011). SwissDock, a protein-small molecule docking web service based on EADock DSS. Nucleic Acids Res..

